# Knowledge, attitudes, and self-reported practices (KAP) towards hand hygiene in medical students versus the public

**DOI:** 10.1007/s11845-022-02918-x

**Published:** 2022-01-13

**Authors:** Patience Mwesigye, Baljot Sekhon, Amit Punni, Gemma McDonnell, Omar Salman, Sarah Hyde, Patrick E. O’Donnell

**Affiliations:** University of Limerick, Castletroy, Limerick, Ireland

**Keywords:** Behaviours, COVID, Hand hygiene, Medical, Practices, Public, Students

## Abstract

**Background:**

The COVID-19 pandemic and its associated morbidity, mortality, and economic disruption has reignited interest in simple protective and preventive measures.

**Aims:**

The aim of this study was to assess the knowledge, attitudes, and practices (KAP) of hand hygiene in a sample of medical students in Ireland and members of the public to evaluate these within the context of the COVID-19 pandemic. We also explored any differences between the two groups.

**Methods:**

A 35-question survey was formulated and circulated to potential participants comprising Irish medical students and members of the public. The data was analysed using Microsoft Excel with *P*-values being calculated using chi-squared goodness-of-fit analysis.

**Results:**

There were 356 responses to the survey, categorised into medical students and general public populations. Incomplete surveys were removed leaving 303 responses. There was no statistical difference between the groups for attitudes and self-reported practices towards hand hygiene. Statistical differences were found between the two groups in terms of knowledge.

**Conclusions:**

The study showed that medical students and the public had a good knowledge base and positive attitude in regards to hand hygiene. Both groups displayed consensus that the practices are essential, especially within the current pandemic context. However, larger studies, involving multiple universities and a larger portion of the public, may be useful to ascertain whether there is a true difference in the KAP between healthcare students and the general public.

## Introduction

The global response to the coronavirus (COVID-19) pandemic has included economic, legislative, and policy actions. The use of masks and social distancing has been recommended to limit the spread of COVID-19. In addition, governments across the world have emphasised the importance of hand hygiene. Hand hygiene is a globally accepted method of reducing the incidence of respiratory infections [1]. It is recognised as an important, and at the same time simple and cost-effective, measure that individuals can take to interrupt the spread of communicable diseases [2]. As a result, there has been an increased interest in hand hygiene from the public. Sales of hand gels have skyrocketed in the past year and alcohol-based hand gels are now present in most stores [3].

Despite evidence that hand hygiene is highly effective in controlling the spread of certain infectious diseases, it has been shown that compliance with hand hygiene measures amongst healthcare workers could be improved. Previous studies have indicated that medical and nursing students have suboptimal levels of knowledge and attitudes about hand hygiene [4–6]. The literature shows a significant amount of research conducted on knowledge, attitudes, and practices (KAP) of hand hygiene amongst health-care professionals, but relatively little is known about hand hygiene KAP amongst the public. The advent of COVID-19 has resulted in a hand hygiene culture change.

The purpose of this study is to assess the KAP towards hand hygiene of Irish medical students and the public. In addition, a special emphasis was placed upon KAP towards hand hygiene in relation to the COVID-19 pandemic.

## Methods

The primary focus of this study was to compare and evaluate knowledge and attitudes towards hand hygiene amongst two chosen populations: medical students and the public.

This study employed a quantitative survey approach, using a standardised questionnaire, adapted from Rosen et al. [7]. The survey was circulated electronically via the Qualtrics platform. The questionnaire was composed of 35 questions with multiple choice ‘yes or no’ questions and Likert scaling options. It compromised 3 sections: Knowledge in Hand Hygiene, Attitudes towards Hand Hygiene, and Self-reported Hand Hygiene practices. Between August and October 2020, medical students enrolled at the University of Limerick were invited to participate in the online survey. Participants were reached via large-group social media accounts (Facebook) comprising of students in graduation years: 2020, 2021, 2022, and 2023. Additionally, the public cohorts were accessed via social media networks including WhatsApp and Twitter. Prior to its distribution, a pilot study was conducted by members of the research team (*n* = 7) to improve the layout, assess completion time, and to allow for minor clarifications of the questionnaire.

Data was analysed using Microsoft Excel. Each population was evaluated by age and demographic (medical student or the public). *P*-values were calculated using chi-squared goodness-of-fit. During data analysis of the “attitudes” questions, ‘agree’ and ‘strongly agree’ response options were combined as ‘agree’ responses, and ‘disagree’ and ‘strongly disagree’ response options were combined to form the ‘disagree’ response. In the analysis of the “self-reported practices” questions, the response ‘Not applicable’ was treated as missing data. ‘Unsure’ was considered a neutral response and it was not combined with any other responses.

This study was approved by The Faculty of Education and Health Sciences (EHS) Research Ethics Committee, University of Limerick. The study was conducted anonymously with no identifiable data recorded. Informed consent was given by participants via tick-box agreement prior to participating in the survey.

## Results

The survey had a total of 356 respondents. Fifty three of these respondents did not complete the survey and thus this data was removed from evaluation. Data evaluated from the adjusted total of 303 respondents comprised of 190 public participants (62.7%) and 113 medical student participants (37.6%).

Demographic questions about geographical location, age group, and prior professional hand hygiene training were collected. Seventy-five percent of the respondents were from Ireland, 19% from Canada, and the rest from England, USA, France, Australia, Dubai, Ghana, and Uganda. The 18–29 age group formed the majority of the respondents (43%), followed by 30–39 (29%), 60+ (13.5%), 50–59 (9.2%), and 40–49 (5%). Ninety-eight percent of the medical student group and 42% of the public group reported having received prior training in hand hygiene.

The results were divided into three sections according to the group of questions being asked:

1. Knowledge in Hand Hygiene
2. Attitudes towards Hand Hygiene
3. Self-reported Hand Hygiene Practices

### Knowledge in hand hygiene

Knowledge in hand hygiene was similar between both groups. Two questions were more likely to be answered correctly by the general public (Q1.1, Q1.2), although this did not reach statistical significance. All of the other questions in this section were more likely to be answered correctly by medical students, although this only reached statistical significance for questions Q1.3, 1.4, 1.5, 1.7, and 1.8 (*P*-value for chi-squared goodness-of-fit < 0.05). Of note, 100% of both groups answered correctly to question 1.9 (Table 1).

**Table 1:** Knowledge in hand hygiene questions comparing medical students and the public shown as percentage of total respondents in each group

**Survey question**	**Correct answer**	**Percentage of each group that answered correctly**	
		**Medical students (%)**	**Public (%)**
**1.1** Cold water should be used for hand washing	False	65	73
**1.2** Hot water should be used for hand washing	True	76	79
**1.3** There is no need to remove watches and bracelets when washing hands	**False**	95*	71*
**1.4** There is no need to remove rings when washing hands	**False**	87*	65*
**1.5** There is no need to wash wrists	**False**	99*	87*
**1.6** Hands need to be washed for at least 15 s	True	85	84
**1.7** Hands need to be dried after washing	**True**	94*	82*
**1.8** Good hand hygiene practices prevent an individual from getting an infection	**True**	91*	81*
**1.9** Hand washing is part of personal hygiene	True	100	100

*Denotes statistically significant data

### Attitudes towards hand hygiene

**Fig. 1:**
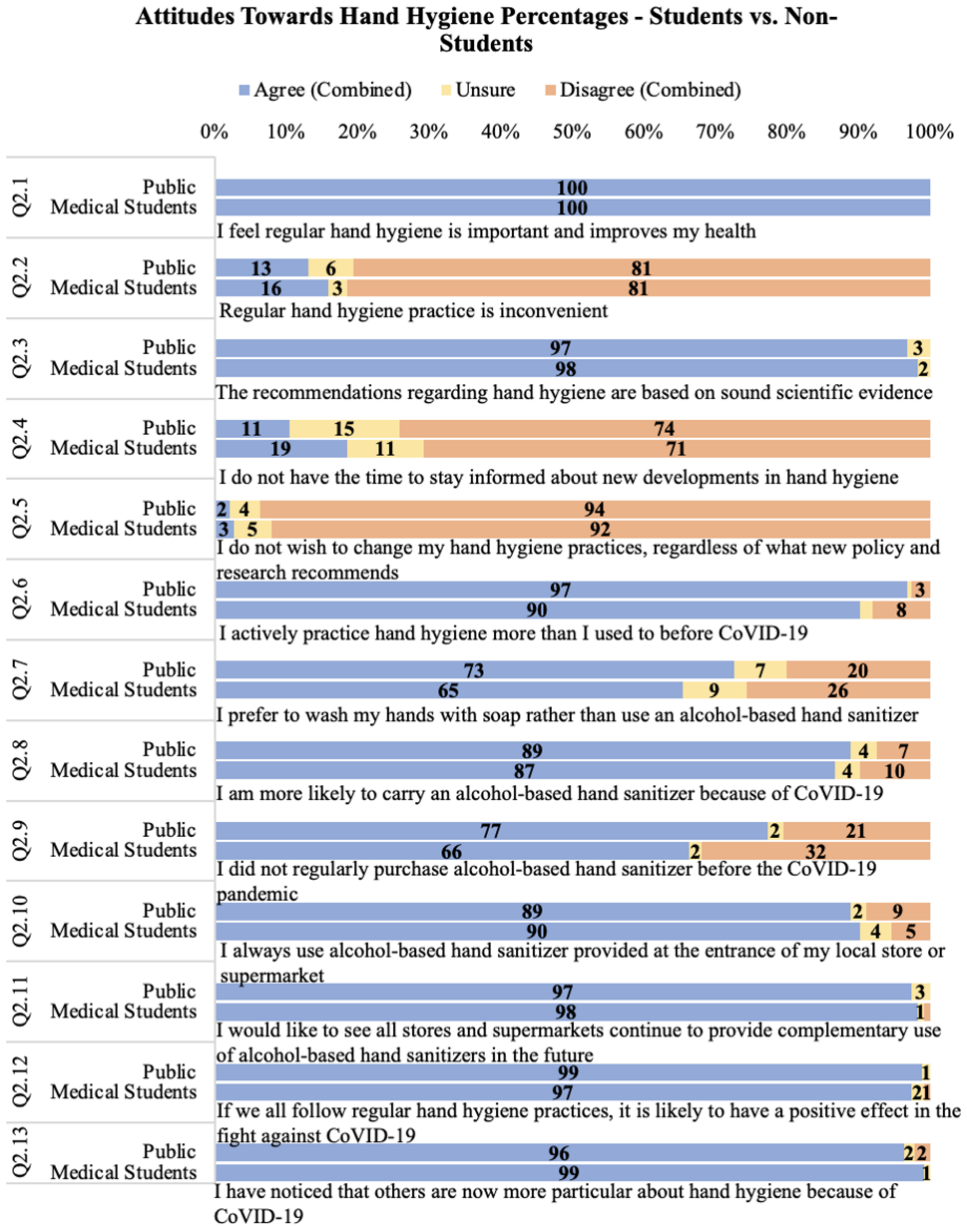
Attitudes towards hand hygiene comparing medical students and the public shown as percentage of total respondents in each group

Overall, there was no significant difference in the attitudes towards hand hygiene between medical students and the public. One hundred percent of respondents agree with the statement ‘I feel regular hand hygiene is important and improves my health’ (Fig. 1).

### Self-reported practices towards hand hygiene

**Fig. 2:**
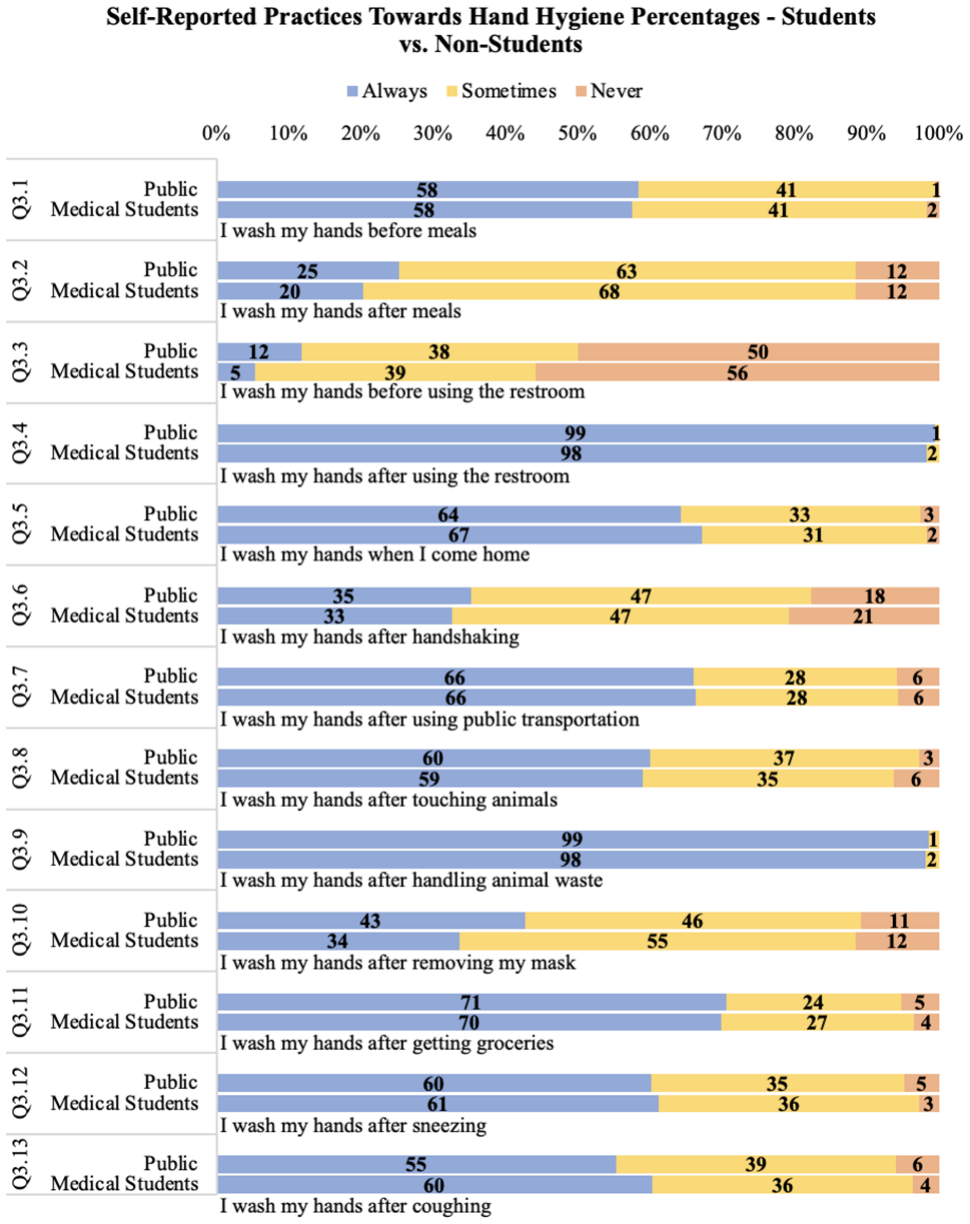
Self-reported practices towards hand hygiene comparing medical students and the public shown as percentage of total respondents in each group

There was no significant difference in self-reported practices towards hand hygiene between the two groups. Question 3.6 is notable as only 33% of the medical student group and 35% of the public group said they ‘Always’ wash their hands after handshaking (Fig. 2).

## Discussion

From this study, medical students and the public participants displayed good knowledge of hand hygiene. In both groups, 97.3% (*n* = 295) participants agreed that recommendations regarding hand hygiene are based on sound scientific evidence.

This is the first study of KAP between medical students and the public on hand hygiene in Ireland. In contrast, there have been recent studies comparing cohorts within the medical profession. A study by Kingston et al. in 2018 looked at attitudes towards hand hygiene in a hospital setting between nursing students and medical students. In accordance with the results of our study, it also showed a general positive outlook on hand hygiene within both groups [4]. The nursing students had a greater hand hygiene compliance compared to medical students; however, there was suboptimal compliance overall.

A study in Saudi Arabia assessed hand hygiene knowledge and compliance amongst undergraduate medical students during their clinical phase of training using the WHO’s “Five Moments for Hand Hygiene”. Compliance was measured during OSCE sessions and only 29% were able to correctly identify all five indications for hand hygiene, indicating a poor knowledge base. Compliance was found to be equally as poor at 17% [5], differing from our study, which had medical students displaying a good knowledge based on hand hygiene. A cross-sectional study in India evaluated hand hygiene knowledge, attitude, and practice amongst medical and nursing students using a WHO hand hygiene questionnaire. In contrast to our study, only 9% of participants had good knowledge regarding hand hygiene. Nursing students knowledge (*P* = 0.023) and attitude (*P* < 0.05) were significantly better than medical students. A German study reported on the beliefs of medical students regarding hand hygiene during their first clinical year in a hospital setting [8]. Overall, only 21% (18/85) of medical students marked all of the indications for hand hygiene correctly, whilst 67% identified WHO’s “Five Moments for Hand Hygiene”. A UK study found that 58% of medical students were unaware of correct indications for alcohol-based hand rub (ABHR) and half of the students reported a perceived lack of teaching on infection control and hand hygiene during their education [9]. These studies suggested a lack of knowledge concerning hand hygiene indications amongst medical students and differed from the results of our study pertaining to medical students’ knowledge.

An interesting topic that emerged was the apparent influence that the COVID-19 pandemic has had on individuals. Of the public participants, 96.8% (*n* = 184) and 90.2% (*n* = 102) medical student participants acknowledged that they now actively practice hand hygiene more regularly than prior to COVID-19. From this, it is clear that the COVID-19 pandemic has had an impact on hand hygiene practices in both groups. This is mirrored in a recent study in France which looked at how hand hygiene behaviour of healthcare workers related to the current dynamic of the COVID-19 pandemic [10].

The knowledge that the public had on hand hygiene was almost equivalent to that of a medical student, possibly due to public health efforts made globally to raise hand hygiene awareness. Nonetheless, there remains a slight gap in knowledge amongst the two groups. This was signified by the responses between the two groups when asked if removing watches and bracelets is required during hand washing. Of the public group 71% (*n* = 135) answered correctly whilst 94.69% (*n* = 98) of the medical student participants answered correctly (*P* < 0.05). These results show that although global awareness of hand hygiene has increased, work remains to further close the gap regarding best practice.

COVID-19 appears to have impacted the two groups’ attitudes. It was interesting to note that there was a relatively significant preference for soap as opposed to alcohol hand gel. This might be attributed to knowledge from both groups or rather the experience of drying effects of some hand gel products. Both groups have actively started to practice hand hygiene more than they used to before COVID-19. Local efforts to reduce transmission of COVID-19 were welcomed by both groups. Of the public participants, 97.36% (*n* = 185) and 98.23% (*n* = 111) medical student participants would like to see all stores and supermarkets continue to provide complementary use of alcohol-based hand sanitisers in the future.

Although reported attitudes were very positive towards hand hygiene, this did not always translate into reported practices. For example only 33.6% (*n* = 38) of medical students and 42.7% (*n* = 79) of the public participants always wash their hands after removing their mask. Lack of hand washing after handshaking was also noted with 32.6% (*n* = 33) of medical students and 35.2% (*n* = 54) of the public indicating that they always washed their hands after handshaking. However, this might be attributed to the fact that since the COVID-19 pandemic, handshaking has been dispensed of in favour of elbow touching or equivalent. Only 60.3% (*n* = 67) of medical students and 55.3% (*n* = 103) of the public always washed their hands after coughing; this was notable as transmission through hands and coughing is known to be associated with transmission of infectious illnesses [11]. Improving compliance with hand hygiene was explored in a recent systematic review of hand hygiene interventions in a clinical setting. It noted that single and multi-modal strategies can achieve a modest–to–moderate improvement in hand hygiene compliance amongst healthcare staff, with net effectiveness of interventions decreasing where baseline compliance was already high. Less information was available on longer term changes in compliance. Reasons for non-adherence with hand hygiene included skin irritation with ABHR, habituation, and staff workload [12].

This study showed that there has been a recent significant change in hand hygiene practices, and both groups themselves noticed a change in practices. With 96.31% (*n* = 183) of the public participants and 99.11% (*n* = 112) of medical student participants noticing that others are now more particular about hand hygiene because of COVID-19. These results indicate that continued awareness and maintaining protocols are likely to be welcomed in an effort to reduce transmission of the current COVID-19 pandemic and any future pandemic [13].

There are limitations to this study. There was a small number of respondents; thus, we cannot be sure that the ‘public’ in this study is truly representative of the wider population. Furthermore, the respondents from the public were most likely people who are interested in infection prevention and control and may not be representative of the general Irish population resulting in selective bias.

The assessment of knowledge did not include the Five Moments of hand hygiene. The students who participated in this study were all University of Limerick medical students, who had undergone the same training regarding hand hygiene. Additionally, participants’ self-reporting on their practices can be seen to have a bias as people know what they should be doing, but we do not know how they are actually behaving. Participants admitting that they did not always wash their hands before eating may indicate that participants answered honestly.

In order to further explore the impact of COVID-19 on knowledge, attitudes, and self-reported practices of hand hygiene, we would suggest evaluating a similar population at different time points with the progression of the COVID-19 pandemic. We could thus evaluate if there is a similar focus on hand hygiene as the global impact of COVID-19 decreases. With the ongoing global vaccination underway for COVID-19, it would be interesting to examine the influence that being vaccinated has on hand washing as well. Another area to explore would be a comparative analysis between different populations of healthcare workers, which might help to show any discrepancies in hand hygiene, and where additional measures and training are needed. Similarly, the desire for face masks going forward both within and outside of the healthcare setting could be evaluated to understand the needs/wants of various populations in a COVID-19 free world.

## Conclusion

Our study shows that both medical students and the public have good knowledge and positive attitudes towards hand hygiene, although self-reported practices may be suboptimal at certain times. This indicates a necessity in including targeted topics towards hand hygiene practices in future educational initiatives especially in the current climate. There is agreement that proper hand hygiene practices play an essential role in combating illness including COVID-19. Continued efforts to maintain adequate hand hygiene practices in both healthcare professionals and the public is essential in controlling the fight against COVID-19 and future pandemics.

## References

[1] Rabie T, Curtis V (2006). Handwashing and risk of respiratory infections: a quantitative systematic review. Trop Med Int Health.

[2] Hand Hygiene - Health Protection Surveillance Centre (2020) https://www.hpsc.ie/a-z/microbiologyantimicrobialresistance/infectioncontrolandhai/handhygiene/. Accessed 18 Dec 2020

[3] Taylor C (2020) Sales of hand sanitizer are skyrocketing due to the coronavirus, leading to rationing and price hikes. https://www.cnbc.com/2020/03/03/coronavirus-hand-sanitizer-sales-surge-leading-to-price-hikes.html. Accessed 18 Dec 2020

[4] Kingston LM, O’Connell NH, Dunne CP (2018). A comparative study of hand hygiene and alcohol-based hand rub use among Irish nursing and medical students..

[5] Al Kadi A, Salati SA (2012) Hand hygiene practices among medical students Interdisciplinary. Perspectives on Infectious Diseases 2012. 10.1155/2012/679129 10.1155/2012/679129 PMC3457633 23024653

[6] Nair SS, Hanumantappa R, Hiremath SG, Siraj MA, Raghunath P (2014) Knowledge, attitude, and practice of hand hygiene among medical and nursing students at a tertiary health care centre in Raichur, India. International Scholarly Research Notices Preventative Medicine 2014. 10.1155/2014/608927 10.1155/2014/608927 PMC4045463 24967144

[7] Rosen L, Zucker D, Brody D, Engelhard D, Manor O (2009). The effect of a handwashing intervention on preschool educator beliefs, attitudes, knowledge and self-efficacy. Health Educ Res.

[8] Graf K, Chaberny IF, Vonberg RP (2011). Beliefs about hand hygiene: a survey in medical students in their first clinical year. Am J Infect Control.

[9] Mann CM, Wood A (2006). How much do medical students know about infection control?.. J Hosp Infect.

[10] Huang F (2021). COVID-19 outbreak and healthcare worker behaviour change toward hand hygiene practices. J Hosp Infect.

[11] World Health Organization (2009) WHO guidelines on hand hygiene in health care: first global patient safety challenge clean care is safer care. Geneva: World Health Organization; 7, Transmission of pathogens by hands. https://www.ncbi.nlm.nih.gov/books/NBK144014/. Accessed Dec 2020 23805438

[12] Clancy C, Delungahawatta T, Dunne CP (2021). Hand-hygiene-related clinical trials reported between 2014 and 2020: a comprehensive systematic review. J Hosp Infect.

[13] Centers for Disease Control and Prevention (2020) Coronavirus disease 2019 (COVID-19): FAQ on hand hygiene. https://www.cdc.gov/coronavirus/2019-ncov/infection-control/hcp-hand-hygiene-faq.html. Accessed Dec 2020

